# Enhancing mesenchymal stem cells cultivated on microcarriers in spinner flasks via impeller design optimization for aggregated suspensions

**DOI:** 10.1186/s40643-023-00707-7

**Published:** 2023-12-03

**Authors:** Botao Zhang, Qiaohui Lu, Gance Dai, Yi Zhou, Qian Ye, Yan Zhou, Wen-Song Tan

**Affiliations:** State Key Laboratory of Bioreactor Engineering, East China University of Science and Technology, Shanghai, 200237 China

**Keywords:** Umbilical cord-derived mesenchymal stem cells, Microcarrier suspension cultivation, Computational fluid dynamics, Spinner flask, Flow field structure, Cell–microcarrier aggregate size

## Abstract

During the ex vivo expansion of umbilical cord-derived mesenchymal stem cells (hUCMSCs) in a stirred tank bioreactor, the formation of cell–microcarrier aggregates significantly affects cell proliferation and physiological activity, making it difficult to meet the quantity and quality requirements for in vitro research and clinical applications. In this study, computational fluid dynamic (CFD) simulations were used to investigate the effect of an impeller structure in a commercial spinner flask on flow field structure, aggregate formation, and cellular physiological activity. By designing a modified impeller, the aggregate size was reduced, which promoted cell proliferation and stemness maintenance. This study showed that increasing the stirring speed reduced the size of hUCMSC-microcarrier aggregates with the original impeller. However, it also inhibited cell proliferation, decreased activity, and led to spontaneous differentiation. Compared to low stirring speeds, high stirring speeds did not alter the radial flow characteristics and vortex distribution of the flow field, but did generate higher shear rates. The new impeller’s design changed the flow field from radial to axial. The use of the novel impeller with an increased axial pumping rate (Q_z_) at a similar shear rate compared to the original impeller resulted in a 43.7% reduction in aggregate size, a 37.4% increase in cell density, and a better preservation of the expression of stemness markers (SOX2, OCT4 and NANOG). Increasing the Q_z_ was a key factor in promoting aggregate suspension and size reduction. The results of this study have significant implications for the design of reactors, the optimisation of operating parameters, and the regulation of cellular physiological activity during MSC expansion.

**Graphical Abstract:**

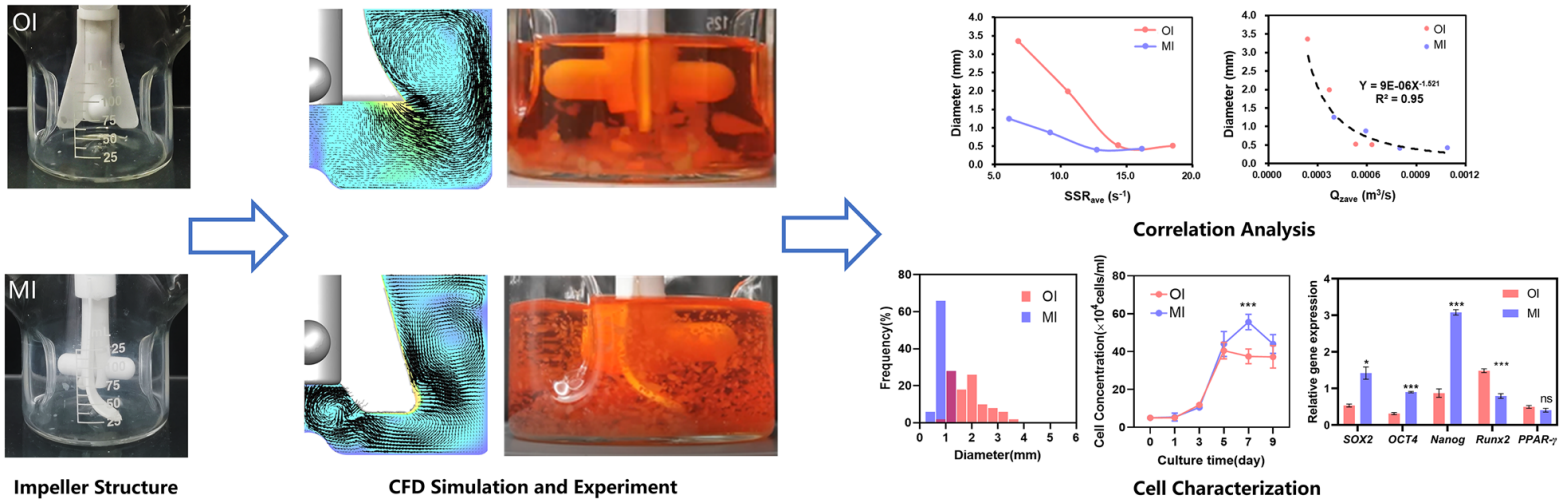

**Supplementary Information:**

The online version contains supplementary material available at 10.1186/s40643-023-00707-7.

## Introduction

Since the initial discovery of human mesenchymal stem cells (hMSCs) in 1968 (Friedenstein et al. 1968), their therapeutic potential in immunological modulation, tissue repair, and regenerative disorders has been widely recognized (Gregoire et al. 2017; Papaccio et al. 2017). Various adult tissues, including bone marrow, adipose tissue, umbilical cord, and skin, can serve as sources of hMSCs (Hoang et al. 2022; Lin et al. 2019). Among these, human umbilical cord-derived mesenchymal stem cells (hUC–MSCs) have garnered significant attention due to their easy access, reduced ethical considerations, low immunogenicity, and robust cytokine secretion capabilities (Barberini et al. 2014; El Omar et al. 2014). Despite their immense potential, the number of hUC–MSCs that can be isolated from a single umbilical cord is limited. Consequently, large-scale in vitro expansion is necessary to meet cell number requirements for a single intravenous injection, which typically ranges from 10^7^ to 10^10^ cells (Huang et al. 2018; Jo et al. 2014; Sharma et al. 2014).

Extensive research has demonstrated that traditional static cultivation systems, typical of cell factories, do not meet the current and future demands of the cell therapy market in terms of scalability, stability, and safety (Eibes et al. 2010; Simaria et al. 2014). Therefore, designing a bioreactor based on microcarrier suspensions has become one of the alternative research directions to achieve the stable large-scale in vitro expansion of hMSCs while maintaining their proliferation and differentiation potential (Bin Hassan et al. 2020; Eibes et al. 2010). Spinner flasks are commonly used as suspension cultivation systems for hMSCs, where hMSCs are cultivated on microcarriers. However, in spinner flasks, the overall flow field is not homogenous, and vortices exist below the impeller. Compared to a bulk fluid flow, the fluid in the centre of the bottom of a spinner flask is almost stagnant, leading to the aggregation and sedimentation of microcarriers and cells in this region (Ghasemian et al. 2020; Kaiser et al. 2013). Cell migration bridging between microcarriers, combined with cell secretions of the extracellular matrix, lead to the adhesion of cells to microcarriers and the formation of aggregates (Mei et al. 2010). As the cultivation time increases, the aggregate size continuous to grow, eventually forming millimetre-sized aggregates. The mass transfer limitations within these aggregates affect cell viability and hinder cell digestion and harvesting, resulting in heterogeneous cell populations upon harvest (Alanazi et al. 2019; Ferrari et al. 2012; Luo et al. 2014; Zeddou et al. 2010). Therefore, enhancing the suspension ability of microcarriers and reducing their deposition at the bottom of spinner flasks has the potential to control aggregate formation, promote cell proliferation, and improve cell quality.

In previous studies, researchers have commonly employed process optimization methods to address the issue of cell–microcarrier aggregation, such as optimizing microcarrier control strategies and adjusting agitation speed. By utilizing microcarrier control strategies, researchers have optimized microcarrier concentrations, added fresh microcarriers during the cultivation process (Ferrari et al. 2012), or selected microcarriers with different densities (Kaiser et al. 2013) to improve particle suspension and reduce aggregation size under the same speed and SSR levels, thereby increasing cell density during harvest. However, improvements in aggregate suspensions remain limited. Increasing the impeller speed in spinner flasks can promote microcarrier suspension and reduce the maximum size of aggregates (Jossen et al. 2016). However, this also increases shear forces (Zhang et al. 2022), leading to compromised hMSCs viability and growth (Nienow et al. 2016), ultimately affecting cell expansion. In addition, hMSCs are sensitive to changes in the microenvironment and can undergo spontaneous differentiation due to intermittent high shear forces (0.42 Pa) during flow, as evidenced by increased expression levels of osteogenesis-related genes. These factors are highly unfavourable for stem cell expansion (Jiao et al. 2022; Liu et al. 2012). Reducing the size of aggregates and minimizing shear forces exerted on cells attached to the microcarrier are crucial to control aggregate size and maintain cell viability and stemness. These factors play a vital role in determining the large-scale in vitro expansion of hMSCs.

The impeller structure affects the suspension of cell–microcarrier aggregates in spinner flasks. Namely, after conducting CFD simulation analyses on the flow field generated by an original impeller at different speeds and sizes within a commercial spinner flask, it was found that the flow field was segmented into multiple vortices in all cases, indicating a pronounced radial flow pattern (Berry et al. 2016; da Silva et al. 2020; Ghasemian et al. 2020). This radial flow pattern may be one of the reasons why the original impeller has a weak suspension ability for aggregates and cannot balance aggregate size and shear forces. Therefore, the impeller structure requires optimization to reduce the number of vortices and transform the radial flow into an axial flow, thereby achieving a strong suspension ability at the same SSR level. The axial pumping rate, Qz, is an important characteristic parameter that describes axial flow. When the swept volume of the impeller accounts for approximately 50% of the total volume, the Qz can be used to quantify the suspension ability of the impeller (Huang et al. 2022). Furthermore, consideration into whether there is a dependency between SSR, Qz, and aggregate size is necessary. Current literature predicts and explains aggregate size and cell culture outcomes based on the distribution of SSR and Kolmogorov length scales generated by different rotational speeds of the original impeller (Ghasemian et al. 2020). However, limited studies have explored the influence of impeller structure and the degree of aggregate suspension on these factors, hindering a more comprehensive exploration of the optimization methods for cell culture processes.

This study initially aimed to investigate the impact of different rotational speeds on the growth, aggregate size, and maintenance of stemness in hMSCs using a commercial spinner flask. The findings of this study revealed that lower rotational speeds (< 45 rpm) resulted in the formation of several millimetre-sized aggregates due to microcarrier deposition, leading to mass transfer limitations and subsequent cell death. On the other hand, higher rotational speeds (> 45 rpm) were found to be unfavourable for cell proliferation and stemness maintenance. These results indicated that regulating cell cultivation solely by adjusting rotational speed is not sufficient; the engineering characteristics of the reactor must be tailored to the specific cell requirements. By conducting CFD simulations to analyse the flow field characteristics inside a commercial reactor, a novel impeller design was developed. This novel impeller aimed to achieve a similar mean shear rate (SSR_ave_) as the original impeller, but with a higher mean pumping rate (Q_zave_) and a flow field structure better suited for aggregate suspension at the same rotational speed. When compared to the original impeller, the application of the novel impeller for hUC–MSCs amplification resulted in a reduction of 67% and 56% in cell aggregate size at 30 rpm and 45 rpm, respectively, while increasing the maximum cell density across all speeds. In addition, the novel impeller design upregulated the expression levels of stemness genes and downregulated the expression of differentiation-related genes, thus reducing spontaneous cell differentiation. Overall, this study combined experimental and simulation data to determine that the impeller’s Q_z_ played a more significant role in determining aggregate size compared to the shear rate. These findings provide valuable guidance to achieve better expansion of high-quality and -quantity MSCs.

## Materials and methods

### In vitro cultivation of hUC–MSCs

Passage 2 hUC–MSCs (Sayer Biologics, HUXUC-01001) were cultivated in DMEM medium (Gibco, 12800082, America) supplemented with 5% platelet lysate and 1% penicillin/streptomycin (Beyotime). Cells were maintained in an incubator at 37 °C and 5% CO_2_, with medium replenished every 2 days. When cells reached 80% to 90% confluency, they were harvested using 0.25% trypsin–EDTA. Cells were cryopreserved at a concentration of 2 × 10^6^ cells/mL and stored in liquid nitrogen. Prior to use, cells were thawed and cultivated in 75 cm^2^ T-flasks. Cells at passages 6 to 8 were used in these experiments.

### Amplification of hUC–MSCs on microcarriers

A 125 mL spinner flask reactor was utilized for all experiments. The inner wall of the spinner flask was coated with 5% silicone oil to prevent microcarriers from adhering to the vessel surface. Following manufacturer’s instructions, 100 mL of phosphate buffer solution and 2.5 g/L Cultispher-S microcarriers were added to each spinner flask, and fully hydrated overnight. After sterilization, the microcarrier was washed with fresh culture medium. hUC–MSCs were digested in T75 flasks with 0.25% trypsin–EDTA to obtain single-cell suspensions. Cells were initially seeded in the spinner flasks at a density of 5 × 10^4^ cells/mL. For the first 8 h after inoculation, intermittent shaking at 45 rpm (5 min shaking followed by 25 min rest) was used to promote cell attachment. To investigate the influence of different stirring speeds on cell–microcarrier aggregates after cell adhesion, four continuous stirring speeds (30 rpm, 45 rpm, 60 rpm, and 75 rpm) were investigated after intermittent stirring. All flask reactors were placed in an incubator at 37 °C and 5% CO_2_ during all experiments.

### Cell proliferation and viability assays

1 mL of cell suspensions were taken from spinner flasks and washed twice with PBS. The microcarrier was incubated overnight in a low permeability crystal violet solution containing 0.1 M citric acid. To determine cell counts, the liquid was thoroughly mixed using a pipette and then transferred to a haemocytometer to count the number of viable nuclei present. Subsequently, cells were stained with the Calcein-AM/PI Live/Dead Cell Double Staining Kit (40747ES80, Yeasen) following the manufacturer’s instructions. Subsequently, cells were examined under a fluorescent inverted microscope to visualize green and red fluorescence.

### Cell–microcarrier aggregation analysis

500 μL of cell suspensions were taken from spinning flasks, and imaged using a Nikon microscope. To accurately determine the size of aggregates formed by cells and microcarriers, the area and diameter of each aggregate were quantified using ImageJ software. Results were compiled to create a statistical map illustrating the size distribution of the cell–microcarrier aggregates under specific cultivation conditions.

### Flow cytometry assay

The surface phenotype of hUC–MSCs was characterized using flow cytometry. hUC–MSCs attached to the cell culture flask or microcarriers were digested using a 0.25% trypsin solution. The resulting cell suspension was collected through a cell strainer. Cells were resuspended in a staining buffer composed of 0.1% NaN_3_ and 1% BSA in PBS. Cells were incubated with different cell surface antibodies for 30 min, including CD14, CD34, CD45, CD73, CD90, CD105, and isotype controls. Flow cytometry analysis was performed using a Beckman CytoFLEX instrument, and data were captured from a minimum of 10,000 cells per sample. FlowJo software was utilized for data analysis to determine the percentage of cells expressing each cell surface antigen.

### RNA extraction and quantitative reverse transcription polymerase chain reaction (qRT-PCR)

Total RNA of hUCMSCs was extracted using TRNzol Universal. The extracted RNA was quantified using a microvolume spectrophotometer. To synthesize cDNA, 1 μg of RNA was added to a reverse transcription premix (11141ES60, Yeasen). qRT-PCR was performed using a BIO-RAD CFX96 fluorescence quantitative PCR instrument. Amplification was carried out for 40 cycles using SYBR Green premix (11184ES08, Yeasen). The relative expression levels of specific genes were calculated using the 2^−△△CT^ method, with triplicates performed for each sample. Gene-specific primers were designed using the Primer-BLAST tool provided by the NCBI.

### CFD simulation

**Fig. 1:**
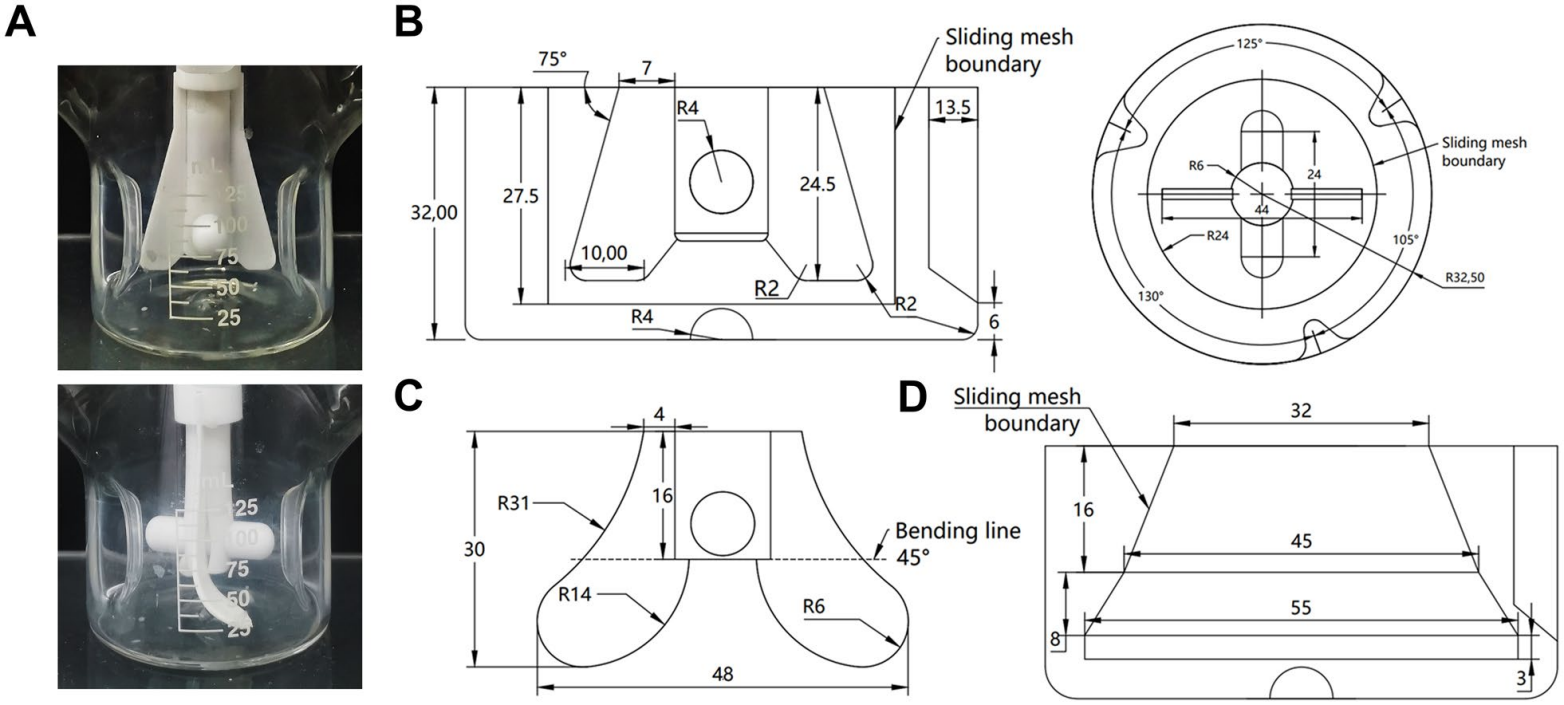
**A** Commercial spinner flask equipped with different impellers. **B** Vertical and horizontal cross sections of the computational domain with the original impellers. **C** Structure and dimensions of the novel impeller before bending. **D** Structural parameters of the computational domain equipped with the novel impeller. All length measurements are in millimetres

This study aimed to simulate and investigate the fluid dynamic characteristics generated by the rotation of two different impellers in commercial spinner flasks. The container and impeller structures are shown in Fig. 1. The simulation settings, including rotational speed and working volume, closely matched the experimental conditions, and a total of 8 operating conditions were simulated.

The fluid flow inside spinner flasks was simulated using water at a temperature of 37℃. The water had a density of 0.998 g/cm^3^, a dynamic viscosity of 7.01 × 10^−4^ ${\text{Pa}} \cdot {\text{s}}$. To accurately simulate the flow patterns inside a bioreactor, the computational domain was divided into two parts: a rotating body known as the rotor, and a stationary body known as the stator. The rotor and stator were not physically separated, and their interaction was measured using the sliding mesh technique. This study primarily investigates the impact of impeller performance on mesenchymal cells, with a solid content of less than 5% that has minimal influence on the overall fluid flow (Kaiser et al. 2013). Therefore, single-phase CFD simulations were conducted in this study.

The mesh for simulations was generated using ANSYS Meshing, employing unstructured grids. Boundary layers were captured using the inflation layer method. The number of elements created varied, ranging from approximately 4 × 10^5^ to 12 × 10^5^ elements, with grid independence confirmed using the impeller torque. All operating conditions were simulated and analysed using approximately 8 × 10^5^ elements.

The two impellers used in these simulations had maximum diameters of 44 mm and 48 mm, respectively. The impeller Reynolds numbers generated at different speeds ranged from 1000 to 3000, indicating that the fluid flow inside the spinner flasks falls within the range of moderate turbulence (Venkat et al. 1996). Turbulence modelling was performed using the *k*–ω SST model (Menter 1994), which takes into account the effects of turbulent shear stress. The standard *k*–ω model was used in the near-wall region, while the standard k–ε model was employed in the bulk liquid region to enhance the accuracy of the simulation results.

The commercial software ANSYS Fluent 2021R1 (ANSYS Inc., Canonsburg, PA, USA) was utilized to determine the governing equations. The coupling between pressure and velocity fields in the flow domain was predicted using the SIMPLE algorithm. The impeller and shaft were modelled as rotating walls, while all other surfaces were considered to have a no-slip boundary condition. Since no deformation of the free surface was observed during the experimental process, the top surface of the model was assigned as a planar wall with zero shear to serve as the gas–liquid contact interface.

Convergence of the simulation was determined based on iteration residuals less than 10^–4^ and a stable impeller torque. The time step was selected to ensure a rotation of the impeller by 0.9° during each step. To obtain velocity and shear rate distributions, all cases were simulated with ten complete revolutions. The temporal-averaged values for velocity and shear rate were calculated based on data from the last four rotations.

### Numerical flow characteristics

The Q_z_ can be characterized by integrating the axial velocity over the cross section at a specific height within the spinner flask. As a result of mass conservation in the flow field, the positive and negative axial pumping rates are equal, giving rise to the following equation:

$$Q_{z} = \mathop \int \limits_{{\Lambda }} V_{Z}^{ + } {\text{d}}A = \mathop \int \limits_{{\Lambda }} V_{Z}^{ - } {\text{d}}A$$

In which, ${V}_{Z}^{+}$ and ${V}_{Z}^{-}$ represent the positive and negative axial velocities, respectively, and A is the cross-sectional area at a specific height within the spinner flask.

By integrating the Q_z_ with respect to the liquid level height (H), the average axial pumping rate (Q_zave_) can be obtained as follows:

$$\begin{aligned}Q_{{{\text{zave}}}} = \frac{1}{H}\int_{0}^{H} {\int\limits_{A} {V_{Z}^{ + } } } {\text{d}}A{\text{d}}H = \frac{1}{H}\int_{0}^{H} {\int\limits_{A} {V_{Z}^{ - } } } {\text{d}}A{\text{d}}H \end{aligned}$$

SSR can be calculated using the velocity components ${V}_{x}$, ${V}_{y}$ and ${V}_{z}$ with the following equation:

$$SSR = \left\{ {\begin{array}{*{20}c} {2\left[ {\left( {\frac{{\partial V_{x} }}{\partial x}} \right)^{2} + \left( {\frac{{\partial V_{y} }}{\partial y}} \right)^{2} + \left( {\frac{{\partial V_{z} }}{\partial z}} \right)^{2} } \right] + } \\ {\left[ {\left( {\frac{{\partial V_{x} }}{\partial y} + \frac{{\partial V_{y} }}{\partial x}} \right)^{2} + \left( {\frac{{\partial V_{x} }}{\partial z} + \frac{{\partial V_{x} }}{\partial x}} \right)^{2} + \left( {\frac{{\partial V_{y} }}{\partial z} + \frac{{\partial V_{y} }}{\partial z}} \right)^{2} } \right]} \\ \end{array} } \right\}^{1/2}$$

### Statistical analyses

Statistical analysis was performed using GraphPad Prism version 8. Data analysis was conducted using *t* tests and one-way ANOVAs. A *p* value less than 0.05 indicated a statistically significant difference between groups.

## Results and discussion

### Effect of rotational speed on the cell–microcarrier aggregate size

**Fig. 2:**
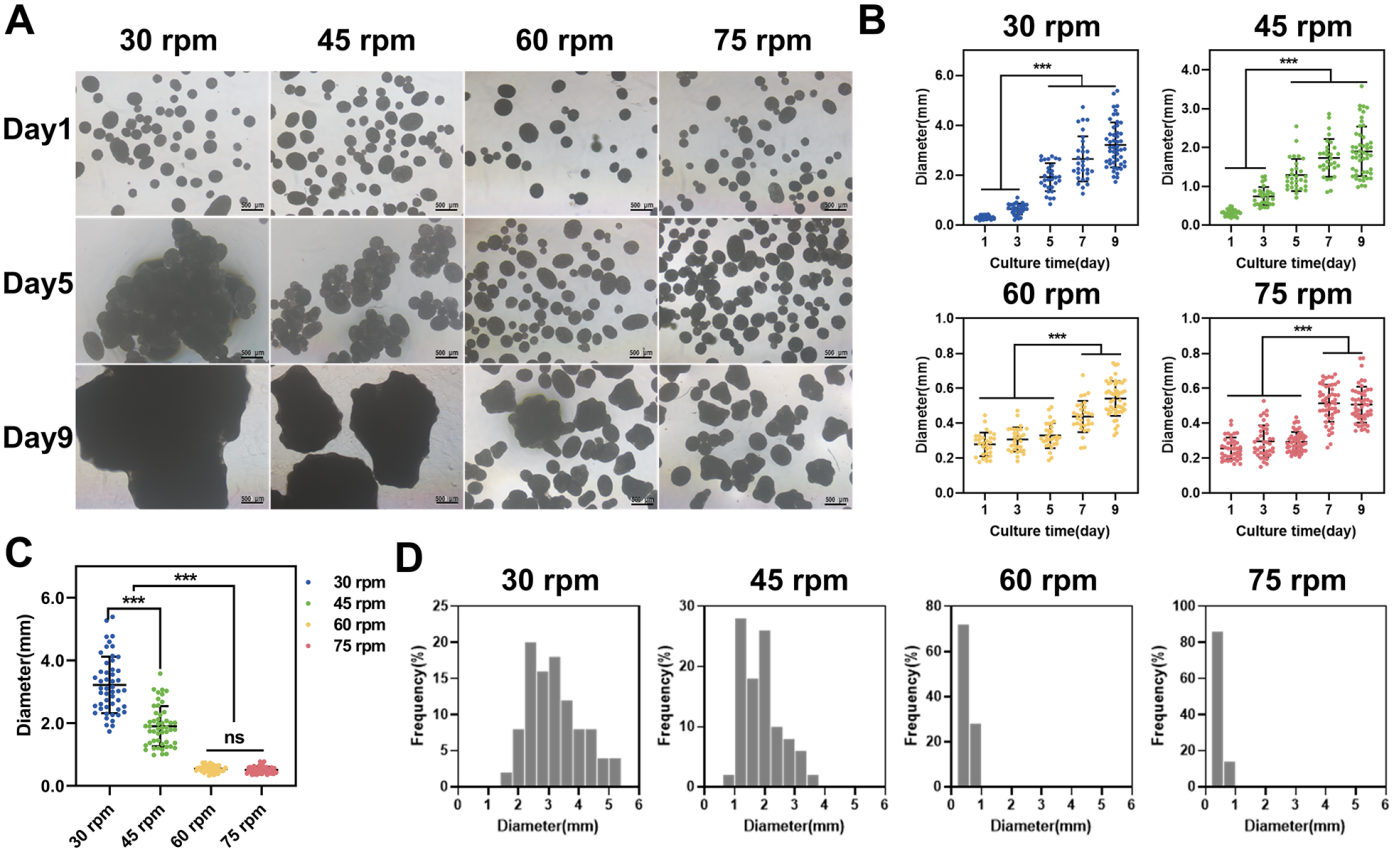
Cell–microcarrier aggregates at different rotational speeds. **A** Microscopic observation of cell–microcarriers cultivated at different rotation rates after 1, 5 and 9 days of cultivation. Scale bar: 500 μm. **B** Changes in cell–microcarrier aggregate size with incubation times. **C** Comparison of aggregate sizes in rotovials at different rotational speeds at day 9. **D** Diameter distributions of cell–microcarrier aggregates

Microcarriers inoculated with cells tend to form aggregates during the cultivation process. In this study, we investigated the effect and pattern of rotational speed on the formation of cell–microcarrier aggregates using four different speeds (30, 45, 60, and 75 rpm) (Fig. 2). On day 1, all microcarriers were dispersed individually in a liquid medium. As cells proliferated and migrated on the microcarrier surface, aggregation was observed. Microcarriers started to adhere and form aggregates at 30 rpm and 45 rpm on day 5, while no significant clustering of microcarriers occurred at 60 rpm and 75 rpm. By the 9th day of cultivation, a large number of cells wrapped around the surface of the microcarriers, forming tight aggregates (Fig. 2A). Quantitative analysis of aggregate size in the culture flasks (Fig. 2B) revealed a significant increase in aggregate size from day 5 onwards at 30 rpm and 45 rpm. However, this phenomenon was not observed until day 7 at 60 rpm and 75 rpm. Higher rotational speeds may have reduced cell migration between microcarriers or decreased their contact frequency due to the increased suspension of microcarriers, thereby slowing down aggregate formation. The average diameter of aggregates formed at the lowest rotational speed was significantly higher at 3.36 ± 0.94 mm compared to the other groups. Similarly, larger aggregates of 1.99 ± 0.69 mm were formed at 45 rpm. Speeds below 45 rpm were considered low speeds, while speeds above 45 rpm were considered high speeds. Significantly smaller aggregates were formed at high speeds compared to low speeds, with diameters of 0.52 ± 0.09 mm and 0.51 ± 0.10 mm, respectively (Fig. 2C). Histograms of diameter distributions further reflected the size and distribution homogeneity of aggregates (Fig. 2D). Aggregates ranging from 1 to 6 mm was observed at 30 rpm, while aggregates ranging from 1 to 4 mm were seen at 45 rpm, indicating a wider distribution range. In contrast, aggregates formed at higher rpm had smaller diameters and a more homogeneous distribution. In summary, the size of cell–microcarrier aggregates during cultivated suspension was influenced by the stirring speed. Larger aggregates were formed at low speeds, while high speeds resulted in smaller and more homogeneous size distributions. There appeared to be a rotational speed threshold between 45 and 60 rpm, at which aggregate size began to stabilize. However, the exact effect of this threshold on cultivated hUC–MSCs warrants further investigation.

### Cell proliferation and function in spinner flasks

**Fig. 3:**
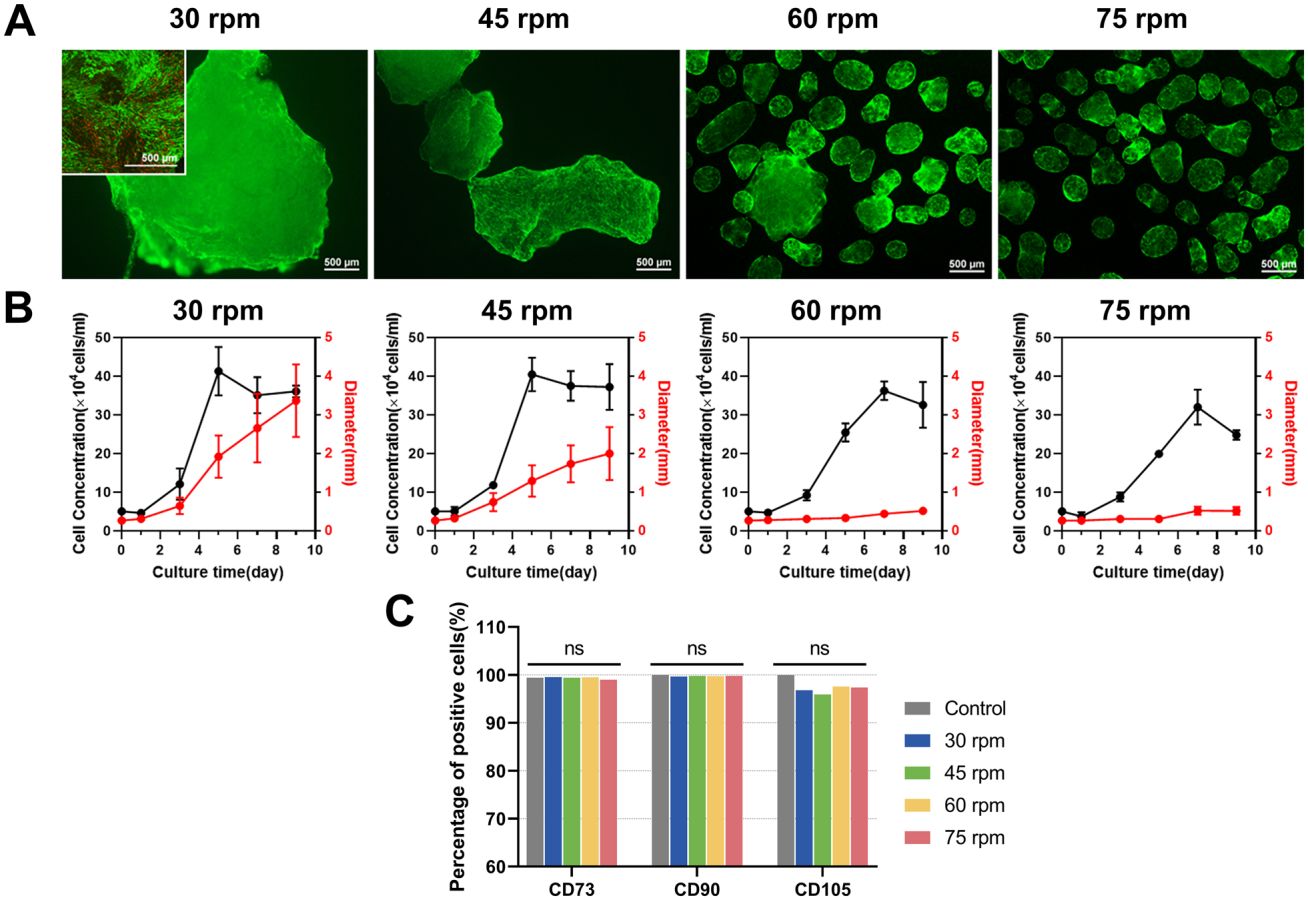
**A** Cell viability staining with Calcein–AM/PI on day 9 under different rotational speeds. Scale bar: 500 μm. **B** Dynamic curves of cell density and aggregate size. **C** Flow cytometry analysis of cell surface markers CD73, CD90, and CD105 at different rotational speeds. The control group consisted of pre-expansion cells

Subsequently, this study evaluated the impact of rotational speeds on cell viability and proliferation. A significant number of viable green fluorescent cells were observed on the surface of aggregates in each group, with minimal red fluorescent dead cells. This indicated that cells on the surface of aggregates exhibited good viability regardless of the rotational speed (Fig. 3A). Upon slicing the 3 mm aggregates formed under 30 rpm, a few dead cells were observed, suggesting a possible decrease in cell vitality within the center of aggregates due to limited substance transfer. Since the aggregates formed under other rotational speeds were smaller than 1 mm, it was difficult to slice them, and there is little possibility of mass transfer limitation. Consequently, cells at the center of aggregates were not visualised. Strategies for cell expansion generally aim to prevent the formation of aggregates larger than 1 mm, since they are prone to diffusion restrictions, sedimentation, and hindered survival of inner cells. Moreover, larger aggregates also impede subsequent cell digestion, separation, and homogeneous suspension in the reactors. As observed in a study by Luo et al. (Luo et al. 2014), the viability of cells significantly decreased beyond 500 μm from the tissue surface when constructing large-sized engineered tissues using Cultispher S. Thus, the formation of aggregates with a diameter smaller than 1 mm is crucial to prevent inner cell death caused by hindering mass transfer.

Figure 3B demonstrates the variation in cell densities and aggregate sizes with cultivation time at different rotational speeds. Under 30 rpm and 45 rpm, the cell density reached its peak on the 5th day, with a respective count of 41.25 ± 5.10 × 10^4^ and 40.45 ± 3.54 × 10^4^ cells/mL before gradually declining. On the other hand, at 60 rpm and 75 rpm, the highest cell density was achieved on the 7th day, measuring 36.20 ± 1.96 × 10^4^ and 31.95 ± 3.68 × 10^4^ cells/mL, respectively, which were lower than the peak densities observed under lower rotational speeds. Analysing the pattern of changes in aggregate size, it appeared that the decrease in cell density at higher rotational speeds could be attributed to two primary factors: (1) the adhesive forces between cells and the extracellular matrix, akin to bonding materials, can cause microcarrier agglomeration, thereby providing more growth space within the aggregates (Caruso et al. 2014). Increased rotational speed reduces microcarrier aggregation, thus partially reducing cell density. (2) Higher stirring speeds intensify fluid flow, leading to increased shear stress on the cells. This excessive shear stress can adversely affect the viability and proliferation of MSCs, and even result in cell detachment from the microcarriers (Maul et al. 2011). Assessing the phenotypic characteristics of expanded cells (Fig. 3C), hUC–MSCs maintained their characteristic surface markers, with CD73 and CD90 positivity rates above 99%, which showed no significant difference compared to pre-expansion levels. Although the CD105 positivity rate remained above 95%, it decreased slightly in comparison with the pre-expansion value. This decline could be attributed to the prolonged enzymatic digestion during the cell harvesting process from the microcarriers and the fluid shear imposed during stirring. Importantly, this reduction in phenotype expression was found to be reversible (dos Santos et al. 2014).

**Fig. 4:**
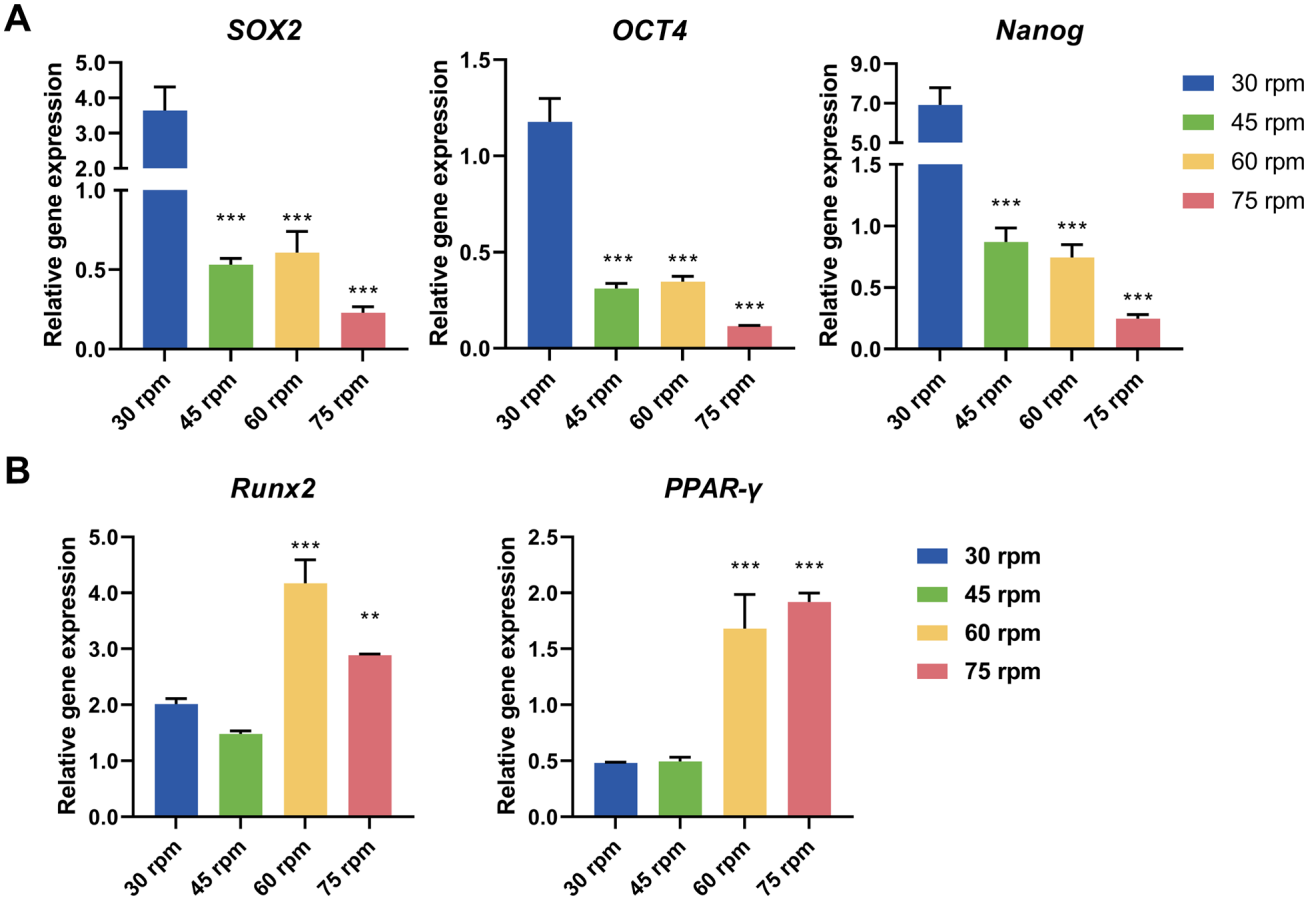
Relative expression levels of pluripotency genes (**A**) and differentiation marker genes (**B**)

The fluid shear generated by the stirring speed affected various biological functions of cells, including cell proliferation and differentiation potential. The expression of stemness genes in cells under different stirring speeds was examined using qRT-PCR. As shown in Fig. 4A, the relative expression levels of stemness markers *SOX2*, *OCT4*, and *Nanog* were highest at 30 rpm. As the stirring speed increased, the relative expression levels of stemness genes significantly decreased. Furthermore, quantitative analysis of differentiation-related genes revealed that the relative expression of *Runx2*, a key regulator of osteogenic differentiation, was lower at 30 rpm and 45 rpm, while it was significantly upregulated with increasing stirring speed. Similarly, the relative expression level of *PPAR-γ*, a key gene involved in adipogenic differentiation, showed an increasing trend with higher stirring speeds (Fig. 4B). In summary, as the stirring speed increased, the stemness of cells cultivated on microcarriers decreased, and spontaneous differentiation occurred. Several studies have reported that fluid shear stress can induce cell differentiation. Compared to static cultivation, the application of shear stress has been shown to promote osteogenic differentiation of MSCs in both 2D and 3D environments (Elashry et al. 2021; Yue et al. 2019). Similarly, appropriate shear stress stimulation is beneficial for the adipogenic differentiation of cells (Adeniran-Catlett et al. 2016). Therefore, shear stress is advantageous in certain differentiation strategies. However, for expansion strategies aimed at harvesting stem cells, shear stress should be minimized to prevent unnecessary differentiation.

The experimental results mentioned above revealed a contradiction between the formation of millimetre-scale aggregates at low stirring speeds and the reduction in stemness associated with high stirring speeds. Although the 30 rpm condition demonstrated better expansion efficiency and superior stemness, the larger aggregates posed difficulties in cell harvesting, often necessitating higher enzyme concentrations and longer digestion times (Caruso et al. 2014). Furthermore, the heterogeneity of cells both inside and outside of the aggregates, especially in terms of differential gene expression, is a concern that needs to be addressed during the expansion of seed cells (Sart et al. 2020). The strategy of reducing aggregate size by increasing the stirring speed fails to meet the requirements of both cell proliferation and stemness maintenance. At 45 rpm, the stemness of cells no longer satisfied the demands of seed cell expansion, and the average aggregate size remained at 1.99 mm, thereby affecting cell digestion. While studies have explored the use of fresh microcarriers to delay aggregate formation, the increased friction and collision resulting from the higher microcarrier concentration can also contribute to cell death (Cherry and Papoutsakis 1986; Sion et al. 2020). Therefore, the goal of this study was to control aggregate size (less than 1 mm) to minimize mass transfer limitations and facilitate cell harvesting while increasing cell density and maintaining higher stemness when compared to the control group. Achieving this required a more in-depth analysis of the fluid dynamics inside the bioreactor using CFD simulations, which can provide valuable insights for developing targeted improvement strategies.

### Simulation of the flow field inside spinner flasks

**Fig. 5:**
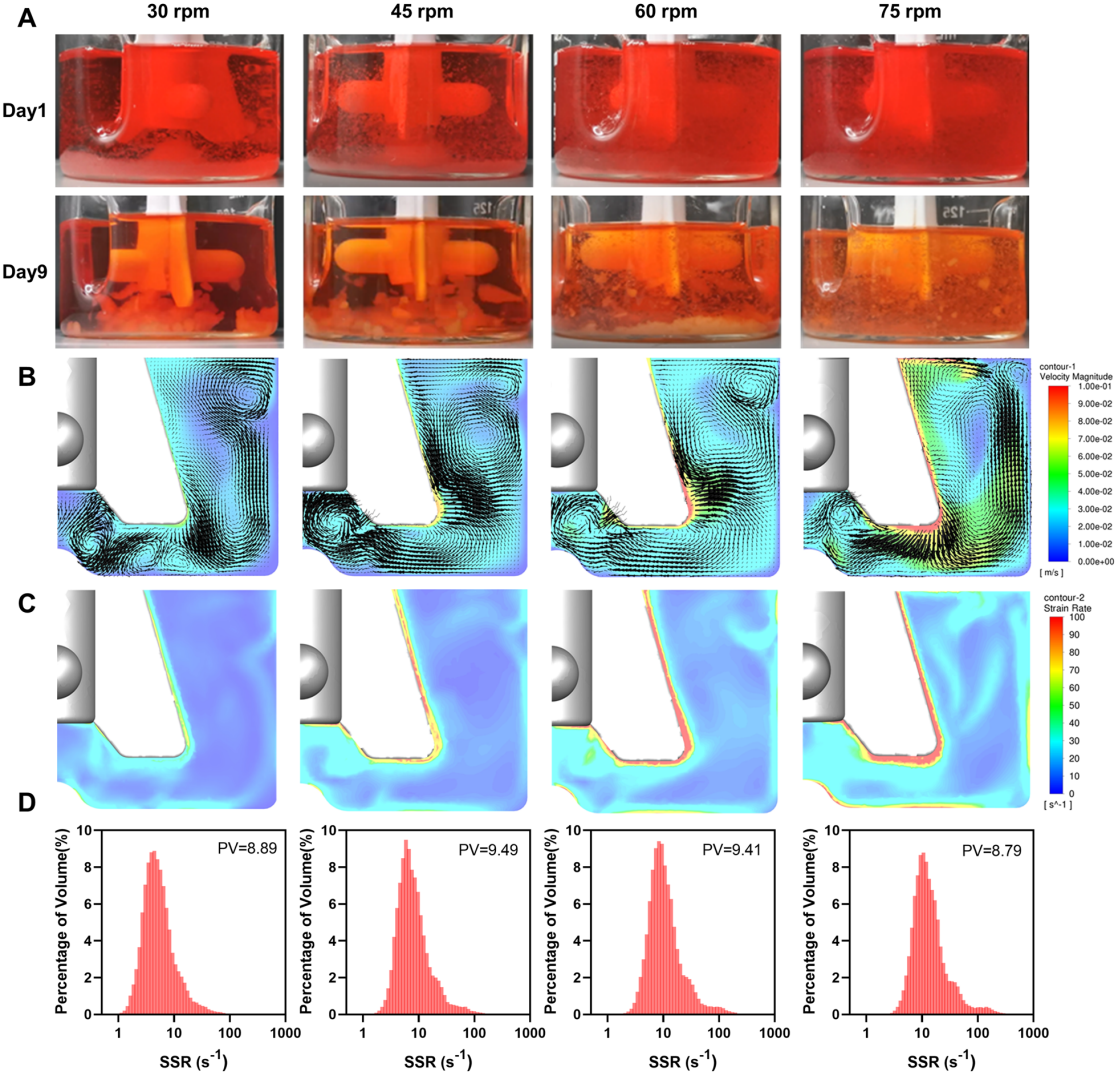
Analysis and simulation of the flow field in spinner flasks equipped with the original impeller. **A** Suspension of aggregates under different rotational speeds on day 9 of incubation. **B** Contour of instantaneous velocity magnitude and vector plots. **C** Contour of SSRs. **D** Distribution of SSR values. PV represents peak value

To gain a deeper understanding of the formation process of microcarrier aggregates, the flow field inside the bioreactor was comprehensively analysed through a combination of experimental observations and CFD simulations (Fig. 5). The distribution of microcarriers during cell expansion could be directly observed visually (Fig. 5A). On the 1st day of cell seeding, no aggregation of microcarriers was observed, and insufficient cell suspension occurred in bioreactors operating at 30 rpm and 45 rpm. A large number of microcarriers settled at the bottom of the bioreactors, especially at the center. By the 9th day, only large aggregates were found to accumulate beneath the impeller, with no suspended microcarrier particles present in the supernatant. At 60 rpm and 75 rpm, the microcarriers at the initial stage of cultivation were well-suspended in the culture medium, but partial particle accumulation was observed in the bioreactor operated at 60 rpm during the later stages of cultivation. These results indicated that sedimentation issues of varying degrees existed in the bioreactor at speeds below 60 rpm. In addition, CFD simulation displayed velocity profiles along the vertical plane of the original impeller at the four different speeds, overlaid with velocity vectors to better observe the distribution and organization of vortices within the spinner flask space (Fig. 5B). Multiple clockwise or counterclockwise vortices were observed within the reactor, primarily located under the shaft and blades, to the right side of the blades, and near the free surface. The positions and sizes of these vortices varied at different speeds but did not decrease in number, consistent with previous reports in the literature (Ghasemian et al. 2020). As expected, maximum velocity was observed at the top of the blades, while the flow velocity was lower at the bottom of the stirrer shaft, with the presence of vortices. The microcarriers may have been trapped in this region, leading to the formation of larger aggregates. A hemispherical bulge was present at the center of the bottom to reduce microcarrier retention, but based on actual observations, microcarrier aggregation could not be reduced at lower stirring speeds.

The results of the SSR contour maps in Fig. 5C were similar to that of the velocity contour maps, with the highest shear rate also occurring at the end of the blades, where they may pose a risk to cells. To further quantify the distribution of shear rates inside the bioreactor, time-averaged shear rate distribution maps for the entire computational domain within four rotations of the impeller were plotted at different speeds (Fig. 5D). As the rotational speed increased, the distribution of SSR moves to higher values. There was no significant difference in the peak shear rate distribution across different speeds, indicating that increasing the speed did not improve the homogeneity of the flow field. This may be due to the similarity in flow field structures at different speeds. Using simulation studies, Berry et al. (Berry et al. 2016) found that particles experienced intermittent shear forces of 0.1 to 0.5 Pa during flow, which may be caused by microcarriers being trapped by vortices beneath blades and intermittently subjected to high shear forces at blade ends.

In a stirred bioreactor, the combined action of fluid circulation and shear forces forms the flow field structure. Macroscopically, in the radial flow field, the fluid is segmented into upper and lower regions by vortices, with stronger local circulation within each region and weaker interactions between regions. This increases the probability of particle collisions and promotes particle aggregation, which is unfavourable for particle suspension. On the other hand, in the axial flow field, the fluid is discharged downwards by the impeller blades, creating overall circulation from bottom to top within the reactor, reducing particle aggregation and favouring particle suspension (Loubiere et al. 2019). The strength of fluid circulation and the degree of particle suspension were strongly correlated and related to the axial fluid velocity, which can be quantified using the Q_z_, as shown below in "Flow behaviour with modified impeller" Section using a comparative analysis. Based on the analysis mentioned above, the flow field generated by the original impeller was similar to the radial flow, with the input energy dissipating into multiple vortices, which is unfavourable for microcarrier suspension cultivation. Adjusting this speed did not change the overall flow field structure, which may also be the fundamental reason for the results obtained in "Cell proliferation and function in spinner flasks" Section. By referring to the characteristics of the Eppendorf Airlift, an axial-flow impeller, the advantages of the original impeller with a high swept volume ratio of the blade were retained, while the end of the blade was extended and modified with an arc to prevent excessive shear forces. In addition, the blades were bent along the horizontal line at the end of the axis to create the modified impeller (MI), which redirected the fluid downwards during impeller rotation, thereby generating overall circulation. To ensure the same input power per unit volume, the diameter of the modified impeller (MI) needed to be slightly larger than that of the original impeller. The specific structural parameters are shown in Fig. 1C and the power number, flow number, and other structural characteristics of the two impellers are presented in Additional file 1: Table S1.

### Flow behaviour with modified impeller

**Fig. 6:**
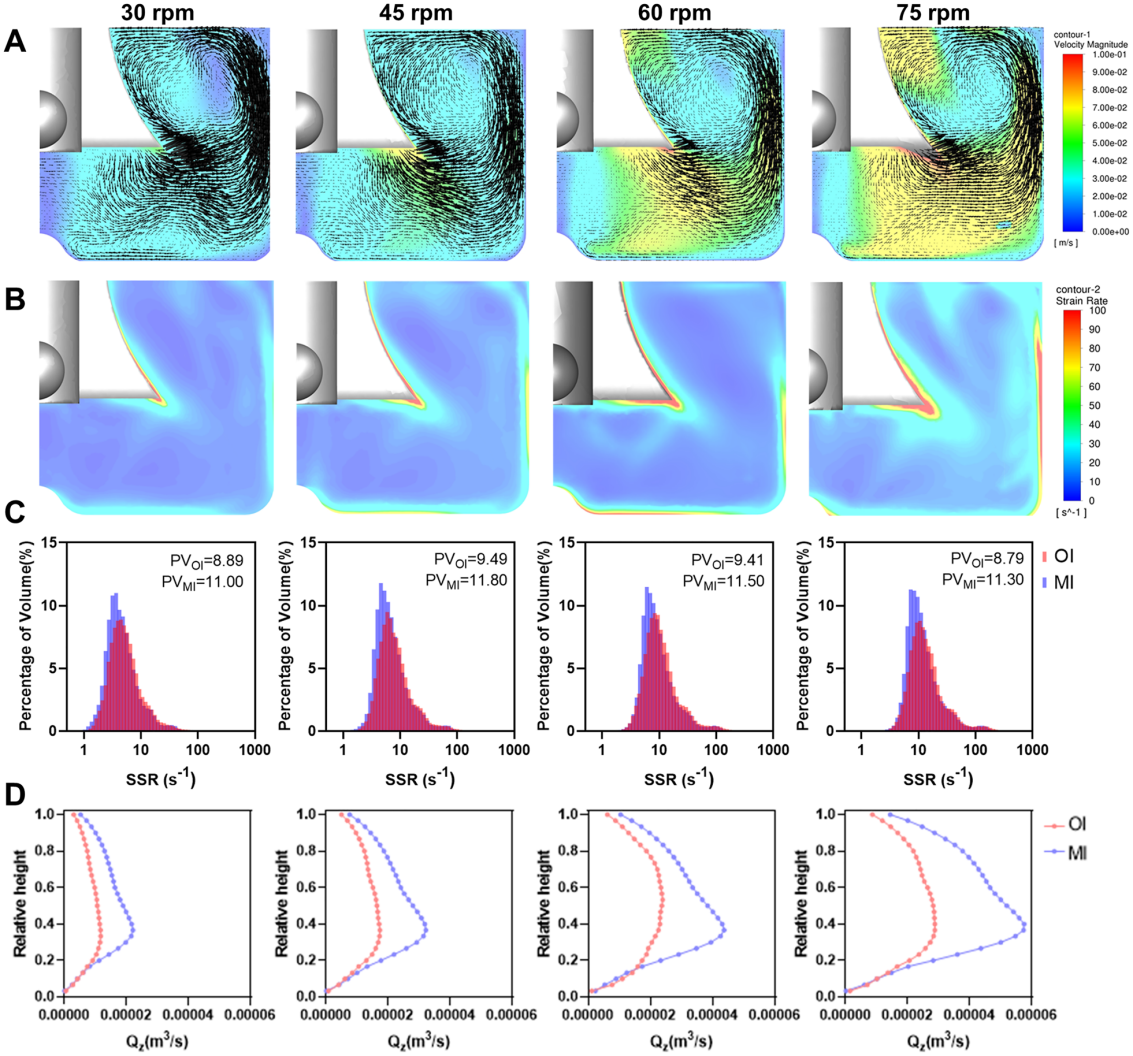
Analysis and simulation of the flow field in the spinner flask equipped with the MI. **A** Contour of instantaneous velocity magnitudes and vector plots. **B** Contour of SSRs. **C** Comparison of SSR distribution between the new and old impellers. PV represents peak value. **D** Comparison of Q_z_ distribution between the new and old impellers

The flow field and shear force distribution generated by the MI at different rotational speeds were simulated using CFD, and the results were compared with the simulated results of the original impeller in "Simulation of the flow field inside spinner flasks" Section. Unlike the original impeller, MI generates a flow pattern that is closer to axial flow: the fluid is discharged downwards by the curved blades of the impeller, colliding with the bottom of the container, and then circulating from bottom to top. Figure 6A shows the flow field structure corresponding to axial flow, where a large vortex with a size close to the liquid level inside the flask dominated the flow, and a weaker small vortex near the bottom of the container was observed. There was no vortex at the bottom of the axis, but the fluid velocity was lower, which may result in the aggregation of microcarriers. Theoretically, in this flow field, microcarriers were more easily suspended and did not remain at the bottom region for an extended period, but this requires validation in subsequent experiments.

Figure 6B illustrates the contour of SSRs generated by the MI, which is similar to the results in "Simulation of the flow field inside spinner flasks" Section. The SSR was highest at the tip of the blades, and the SSR of the bulk liquid was two orders of magnitude lower than the maximum SSR. Table 1 summarizes the SSR_ave_ for both impellers at different rotational speeds. Since the input power per unit volume was substantially similar (Additional file 1: Table S1), the SSR_ave_ of the original impeller was only approximately 15% higher than that of the MI at the same rotational speed. However, the distributions of SSR for the two impellers differed slightly (Fig. 6C). When the SSR was lower than the SSR_ave_, the volumetric contribution of SSR was higher for the MI, indicated by a higher volume percentage (PV) value, suggesting that the homogeneity of the flow field created by the MI was slightly better than that of the original impeller.

**Table 1:** SSR_ave_ and Q_zave_ of two impellers at various rotational speeds

Rotate speed (rpm)	30	45	60	75
SSR_ave_-OI (s^−1^)	6.75	10.54	14.37	18.50
SSR_ave_-MI (s^−1^)	6.06	9.19	12.72	16.16
Q_zave_-OI (m^3^/s)	2.40E-04	3.71E-04	5.33E-04	6.32E-04
Q_zave_-MI (m^3^/s)	4.00E-04	5.93E-04	7.99E-04	10.9E-04

OI represents the original impeller, and MI represents the modified impeller

The structural changes in the impeller had no significant impact on the distribution of the SSR but greatly enhanced the Q_z_. Figure 6D illustrates the distribution of the Q_z_ at different relative liquid levels. For both impellers, an increase in rotational speed led to an improvement in the Q_z_ at various heights. The highest Q_z_ values were concentrated around relative heights of 0.2 to 0.4, which corresponded to the lower-middle section of the original impeller and the region below the bending line of the MI. The Q_z_ values at the liquid surface and bottom of the container were the smallest. However, at the same rotational speed, the Q_z_ generated by the MI is higher at different relative heights when compared to the original impeller, with the maximum value being approximately twice as high. In addition, the Q_zave_ was 50% to 60% higher for the MI. The Q_zave_ generated by the MI at 45 rpm was comparable to that of the original impeller at 75 rpm. This indicated that the MI could suspend aggregates more uniformly at lower levels of SSR, which contributed to investigate the main factors influencing aggregate size.

### Application of the MI to cell cultivation

**Fig. 7:**
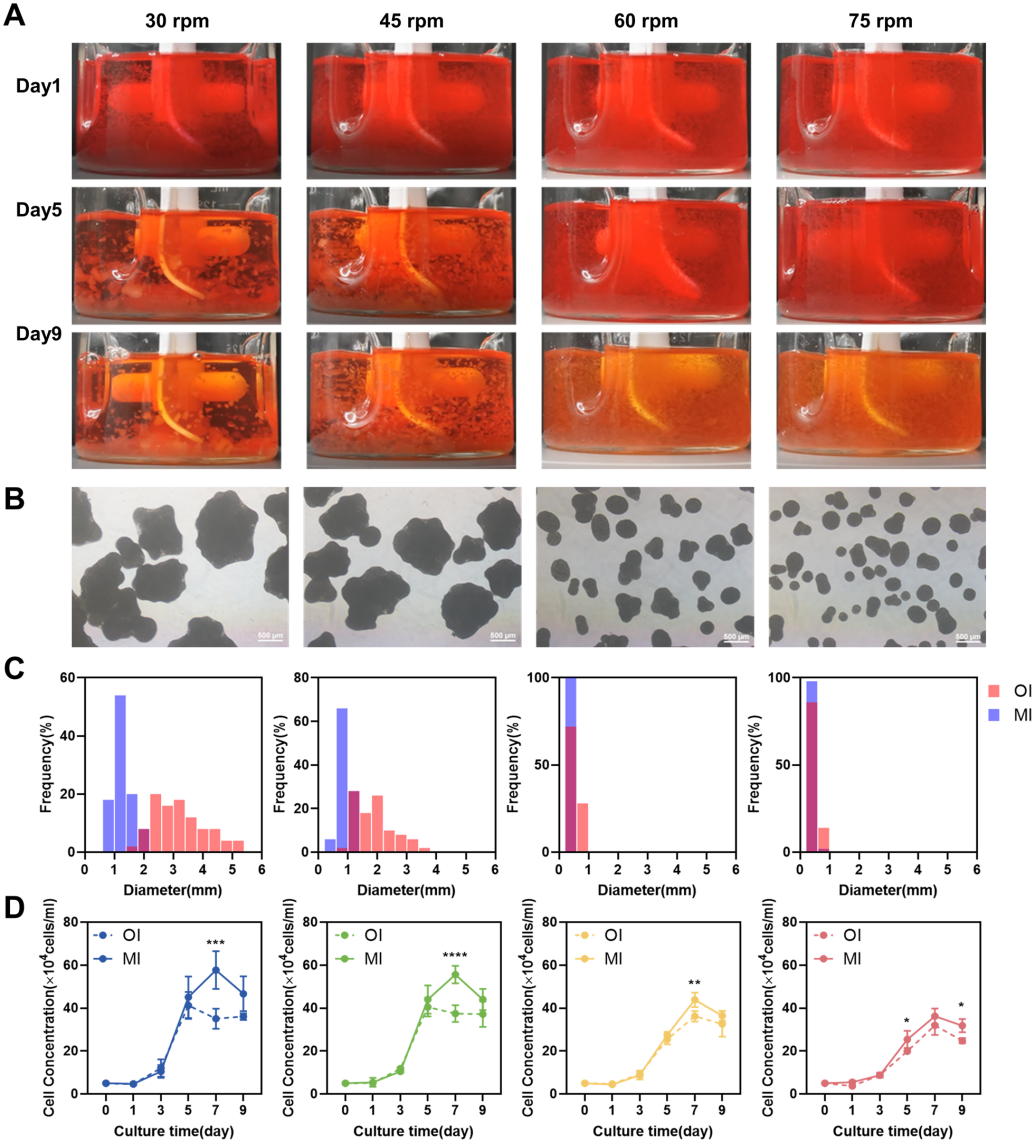
Influence of the MI on cell–microcarrier aggregate size and cell proliferation. **A** Suspension and distribution of microcarriers in flasks at days 1, 5, and 9 of cultivation. **B** Microscopic brightfield images of cell–microcarrier samples on day 9. Scale bar = 500 μm. **C** Comparison of the frequency distribution of cell–microcarrier aggregate diameters before and after impeller modification. Red: original impeller (OI), blue: modified impeller (MI). **D** Cell proliferation curve before and after paddle leaf modification

The MI was employed to cell cultivation. It was observed that the flow pattern created by the impeller was more suitable for the required microenvironment to expand hUC–MSCs, although further validation is necessary to confirm this finding. Under conditions similar to previous studies, the suspension capability of the new impeller was visually assessed in a spinner flask (Fig. 7A). Even at the lowest rotational speed of 30 rpm, the MI demonstrated the ability to effectively suspend microcarriers with no obvious deposition at the bottom of the flask on the 1st day of cultivation. However, by the 5th day, an increase in microcarrier clustering was evident at 30 rpm and 45 rpm, with aggregate deposition observed at 30 rpm. By the 9th day, the majority of microcarriers were distributed in the lower half of the flask, although the suspension achieved with the MI was still superior to that achieved with the original impeller stirring. The MI at 45 rpm and higher rotational speeds ensured optimal suspension of the microcarriers. The quantification of the degree of aggregate suspension using the Q_z_ was found to be feasible.

Furthermore, the impact of the improved impeller stirring on the size of microcarrier aggregates was evaluated. After 9 days of MI cultivation, although dense aggregates surrounded by cells were still present, their size was significantly smaller compared to those formed under the original impeller conditions (Figs. 2A and 7B). The diameter distribution of aggregates formed by the original and improved impellers demonstrated that the average aggregate diameter at 30 rpm decreased from 3.36 ± 0.94 mm to 1.25 ± 0.26 mm, representing only 37% of the original size. Similarly, the average diameter of aggregates at 45 rpm decreased from 1.99 ± 0.68 mm to 0.87 ± 0.18 mm. Under high-speed conditions of 60 rpm and 75 rpm, the diameter consistently remained below 0.5 mm. It is worth noting that as well as reducing the size of microcarrier aggregates, the distribution of aggregate sizes formed under the new impeller stirring was more uniform. These results indicated that optimizing the impeller structure to enhance aggregate suspension could effectively decrease the size of aggregates and promote a more homogeneous distribution.

**Fig. 8:**
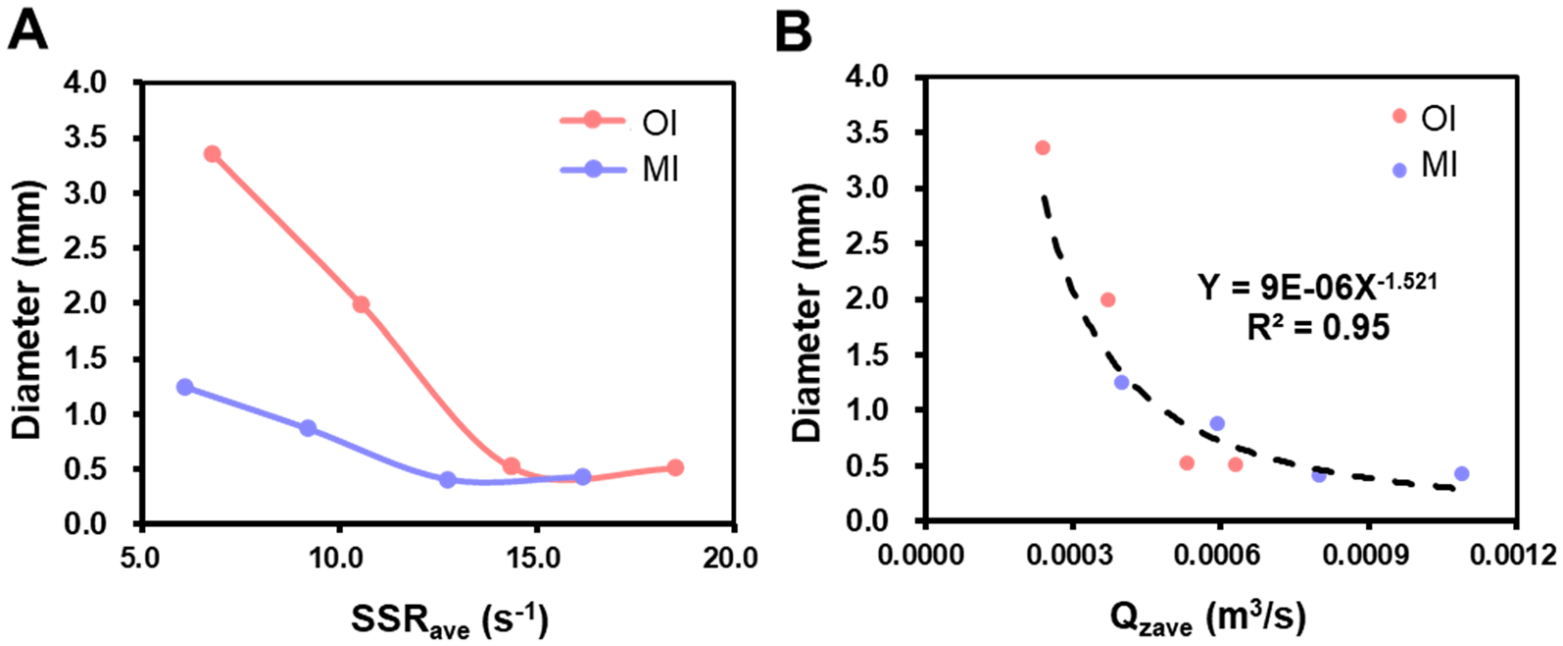
Effect of SSR_ave_ (**A**) and Q_zave_ (**B**) on the average size of aggregates

These experimental results were further analysed by correlating the average aggregate size under different conditions with the simulated values of SSR_ave_ and Q_zave_, to determine the crucial physical factors influencing aggregation. In previous studies, the size distribution of multicellular spheroids was often explained using the Kolmogorov vortex length, wherein the aggregate size is slightly smaller than the vortex length. The shear forces exerted by the vortices were considered the dominant factor influencing aggregate size (Ghasemian et al. 2020). The cell–microcarrier aggregates have a size range of 200–5000 μm, which is much larger than the distribution of Kolmogorov vortex length within the spinner flask (100–300 μm). Therefore, this method is not suitable for this study. In addition, cell and microcarrier aggregates tend to accumulate at the bottom of the spinner flask at lower rotational speed, resulting in increased aggregate size. Hence, the degree of suspension should also be considered as one of the criteria for predicting aggregate size. Shear stress rate (SSR_ave_) and suspension quality index (Q_zave_) were quantified to represent the shear force and degree of aggregate suspension, respectively, and were plotted against the average aggregate size (Fig. 8A, ).

As illustrated in Fig. 8A, the average size of aggregates remained relatively constant once SSR_ave_ exceeded 14 s^−1^. However, when SSR_ave_ was lower than 14 s^−1^, significant differences in average aggregate size were observed between two impellers, indicating that SSR is not the sole determining factor for aggregate size. Figure 8B depicts these data points were fitted with a power-law curve, indicating that Q_zave_ is the dominant factor in determining aggregate size. Notably, when the Q_zave_ fell below 6 × 10^–4^ m^3^/s, the aggregates were not fully suspended (Figs. 5A and 7A), and their average size significantly decreased as the Q_zave_ increased. These findings indicated that in situations, where aggregates were unevenly suspended, their average size was primarily determined by the degree of suspension, while shear forces play a minor role in this context. Overall, these results provide valuable guidance for expansion strategies of hUC–MSCs using microcarrier-based approaches. However, it is also important to consider other factors such as cell quantity and quality when evaluating the effectiveness of hUC–MSCs expansion. Comparing cell proliferation curves before and after modifying the impeller, it was observed that cell density increased under all rotational speeds, reaching its peak on the 7th day (Fig. 7D). The most notable growth occurred at lower speeds, with the highest densities at 30 rpm and 45 rpm increasing from 41.25 ± 5.10 × 10^4^ and 40.45 ± 3.54 × 10^4^ cells/mL to 57.71 ± 7.16 × 10^4^ and 55.58 ± 3.31 × 10^4^ cells/mL, respectively. These results indicated that when the SSR_ave_ was less than 14 s^−1^, the inhibitory effect of shear forces on cell growth was weaker. Furthermore, the improved suspension capability of aggregates reduced their size, which may alleviate mass transfer limitations and enable an increase in cell quantity. However, under high-speed conditions, there was no significant improvement in the maximum cell density before and after impeller optimization. This could be attributed to the inhibitory effect of high SSR generated at high speeds on cell proliferation. The results of cell proliferation provide further evidence of the superiority of the MI in the expansion process of hUC–MSCs.

**Fig. 9:**
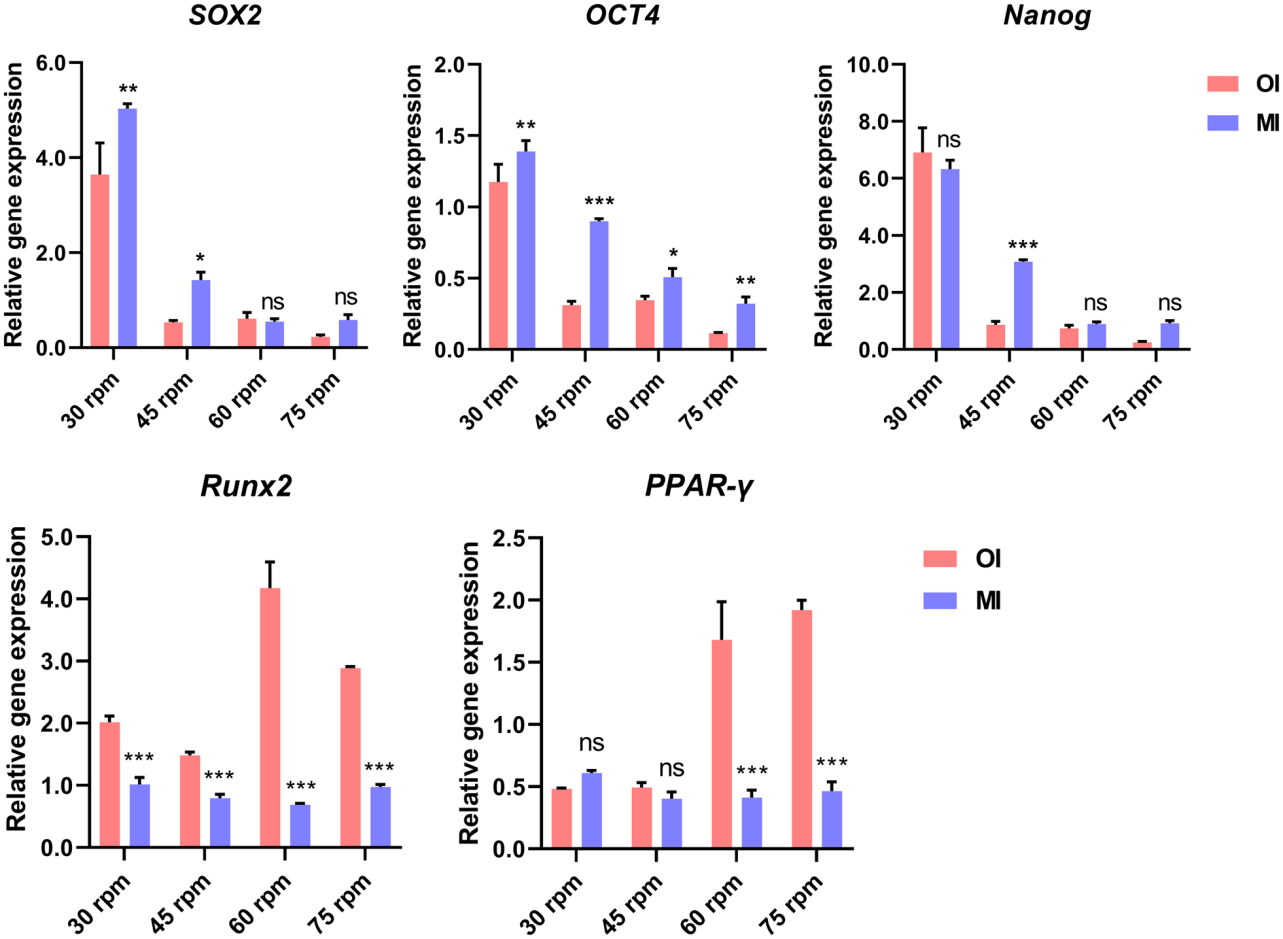
Influence of the MI on the expression of pluripotency (**A**) and differentiation genes (**B**) in cells

To investigate the potential impact of the MI on the differentiation potential of expanded cells, the expression of pluripotency genes and differentiation markers was evaluated (Fig. 9). The qRT-PCR results revealed that the use of the MI led to a significant upregulation of *SOX2*, *OCT4*, and *PPAR-γ* expression in MSCs at 30 rpm and 45 rpm. Under high-speed conditions, only *OCT4* expression was upregulated, while no significant differences were observed in *SOX2* and Nanog expression (Fig. 9A). Moreover, in comparison with the cultivation conditions with the original impeller, the utilization of the MI resulted in an overall reduction of the expression of the osteogenic differentiation regulatory gene *Runx2* and downregulation of the expression of the adipogenic differentiation gene *PPAR-γ* under high-speed conditions (Fig. 9B). The upregulation of pluripotency genes and downregulation of differentiation genes suggest that the MI was less likely to induce spontaneous cell differentiation, and the expanded cells maintained a robust level of stem cell pluripotency. The contrasting gene expression results indicated that both mass transfer limitations and shear forces influenced the maintenance of cell pluripotency. The enhanced shear forces inevitably led to the downregulation of pluripotency genes. However, an increase in the Q_z_ facilitated improved suspension of aggregates, resulting in smaller aggregate sizes and alleviating mass transfer limitations, thus partially counteracting the negative impact of shear forces on pluripotency gene expression. Overall, when considering factors, such as highest cell density, aggregate size, and pluripotency maintenance, it was crucial to have a SSR_ave_ below 14 s^−1^ and a Q_zave_ above 6 × 10^–4^ m^3^/s to achieve optimal conditions. Among these, only the MI with a rotational speed of 45 rpm fulfilled these criteria.

## Conclusion

Controlling the size of cell–microcarrier aggregates and limiting shear forces is often a challenging task in microcarrier-based hUC–MSCs cultivation. In this study, using CFD simulations, the flow field structure was identified as a critical factor affecting aggregate formation in stirred bioreactors. To promote aggregate suspension and control their size, the axial pumping rate Qz was increased. Based on these findings, a new impeller design was developed to improve aggregate suspension. As a result, the average size of aggregates generated during cell expansion was stabilised at approximately 0.87 mm, resulting in a nearly 40% increase in cell density while reducing spontaneous differentiation caused by shear forces. These research findings provide new insights to guide the optimisation of reactor design and operating parameters at different scales, and offer new ways to establish hUC–MSC expansion processes, ultimately contributing to further improvements in cell expansion scale.

### Supplementary Information

**Additional file 1.** Original and modified impeller performance parameter comparison.

## Data Availability

Data will be made available upon reasonable request.

## Abbreviations

hUC–MSCs: Human umbilical cord-derived mesenchymal stem cells
CFD: Computational fluid dynamics
hMSCs: Human mesenchymal stem cells
SSR: Shear strain rate (s^−^^1^)
Q_z_: Axial pumping rate (m^3^/s)
SSR_ave_: Mean shear strain rate across the entire spinner flask zone (s^−^^1^)
Q_zave_: Mean axial pumping rate across the entire spinner flask zone (m^3^/s)
qRT-PCR: Quantitative reverse transcription polymerase chain reaction
${V}_{Z}^{+}$: Positive axial velocity (m/s)
${V}_{Z}^{-}$: Negative axial velocities(m/s)
A: Cross-sectional area at a specific height within the spinner flask (m^2^)
H: Liquid level height (m)
${V}_{x}$, ${V}_{y}$ and ${V}_{z}$: Velocity components (m/s)
OI: Original impeller
MI: Modified impeller
