# Supplementary material for: Enhancing mesenchymal stem cells cultivated on microcarriers in spinner flasks via impeller design optimization for aggregated suspensions
Source: Bioresour Bioprocess. 2023 Dec 3;10(1):89. doi: 10.1186/s40643-023-00707-7 (PMC10992254; doi:10.1186/s40643-023-00707-7)
Supplement: Supplementary file 1 — Additional file 1. Original and modified impeller performance parameter comparison. [file 40643_2023_707_MOESM1_ESM.docx]

Online Additional Material

**Table S1.** Unit volume power input (P/V), power number (N_p_), flow number (N_q_), and pumping efficiency (η) of the two types of impellers at varying rotational speeds.

| Rotate speed (rpm) | 30 | 45 | 60 | 75 |
| --- | --- | --- | --- | --- |
| P/V-OI (W/m^3^) | 0.31 | 1.03 | 2.27 | 4.23 |
| P/V-MI (W/m^3^) | 0.24 | 0.74 | 1.76 | 3.38 |
| N_p_-OI | 1.50 | 1.48 | 1.38 | 1.32 |
| N_p_-MI | 0.75 | 0.69 | 0.69 | 0.68 |
| N_q_-OI | 5.63 | 5.80 | 6.25 | 5.93 |
| N_q_-MI | 7.24 | 7.15 | 7.23 | 7.88 |
| η-OI | 3.74 | 3.93 | 4.54 | 4.51 |
| η-MI | 9.70 | 10.33 | 10.43 | 11.57 |

The power number (N_p_), flow number (N_q_), and pumping efficiency (η) are calculated using the following equations:

$$N_{p}=\frac{P}{\rho N^{3}D^{5}}$$

$$N_{q}=\frac{Q_{zave}}{ND^{3}}$$

$$\eta=\frac{N_{q}}{N_{p}}$$

where P represents the power input from the impeller into the fluid (W), ρ denotes the density of the fluid (kg/m^3^), N signifies the rotational speed of the impeller (rps), and D represents the maximum diameter of the impeller (m). Higher η indicates that the impeller has a higher axial pumping capacity while generating the same level of SSR_ave_.
